# Unrolling of *Syngonium podophyllum*: Functional Anatomy, Morphology and Modelling of Its Peltate Leaves

**DOI:** 10.1002/adbi.202500279

**Published:** 2025-09-04

**Authors:** Michelle Modert, Nick Seinsche, Sören Bartels, Tom Masselter, Thomas Speck

**Affiliations:** ^1^ Plant Biomechanics Group @ Botanic Garden University of Freiburg Freiburg Germany; ^2^ Cluster of Excellence livMatS @ FIT—Freiburg Center for Interactive Materials and Bioinspired Technologies Freiburg Germany; ^3^ Department for Applied Mathematics University of Freiburg Freiburg Germany

**Keywords:** cell expansion, leaf unfolding, mathematical model, peltate leaves

## Abstract

The mechanisms underlying leaf unfolding remain largely speculative and are often inferred from mathematical models. Peltate leaves, unlike typical foliage leaves, frequently emerge in a “rolled‐up” state. This study investigates mechanisms related to the unrolling process in the peltate species *Syngonium podophyllum* by analyzing anatomical and morphological changes and quantifying forces that arise during unrolling. Leaf unrolling in *S. podophyllum* appears to be primarily driven by cell expansion, particularly in the upper epidermis, with cell turgor playing an important role in leaf development and unrolling. Considering leaf properties such as cell dimensions and leaf radius of curvature, this work proposes a mathematical model to further characterize the unrolling process. The model provides satisfactory predictions of curvature variations, highlighting its potential for other plant movements involving dynamic curvature changes.

## Graphical abstract

The unrolling of the peltate leaves in *Syngonium podophyllum* is analyzed and quantified (left‐hand side to center). These measurements serve to verify a mathematical model for leaf unrolling based on the model used in Schmidt (2007). An additional formula for obtaining a layer mismatch from a prescribed radius is derived.

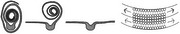

## 1 Introduction

Leaf unfolding, also known as leaf emergence or leaf expansion, is a crucial process in plant growth and development. Despite its importance for capturing sunlight and exchange of gases, the process of leaf unfolding is not yet thoroughly understood. This gap in understanding has led to research focusing on specific plant species and the development of mathematical models to describe the unfolding process.^[^ ^1^, ^2^ ^]^ Such studies often aim to predict leaf unfolding under various conditions, yet they remain limited in their scope and applicability. Even the most well‐known compliant structures, namely the deployable solar panels or antennae used in satellites,^[^ ^3^, ^4^ ^]^ which are often discussed in relation to leaf and flower folding and unfolding patterns, are inspired by traditional Japanese folding techniques (Origami) more than by plants. Our goal is to address these limitations and develop a more comprehensive understanding of the leaf unrolling process for *Syngonium podophyllum*.

Similar to flower opening in many plant species, leaf unfolding is presumably driven by differential growth rates on the inner and outer side of the plant surfaces and/or reversible turgor changes.^[^ ^5^, ^6^, ^7^ ^]^ In some Poaceae, bulliform cells are responsible for motion. These specialized cells form in the multiple folds of grass lamina and provoke a hinge‐like opening movement when they expand due to increasing turgor and vice versa.^[^ ^8^, ^9^ ^]^ Hinge cells can also be found in leaves of the palm family (Arecaceae), where they lead to folding in case of dehydration.^[^ ^10^ ^]^

For simple angiosperm leaves, unfolding patterns can be numerically described by taking leaves as plane surfaces with straight parallel folds.^[^ ^1^, ^11^ ^]^ For ribbon‐shaped undulating monocot leaves and blades of kelp, the models predict edge actuation attributable to differential growth rates of the leaf edge and center.^[^ ^12^ ^]^ In adult leaves, the major veins strengthen the lamina due to increased mechanical properties compared to the surrounding tissue.^[^ ^13^, ^14^, ^15^, ^16^ ^]^

Another well‐known leaf movement, which, however, serves as a defense against herbivores, is the closure of *Albizia julibrissin* or *Mimosa pudica* leaflets caused by mechanical stimuli. Ion and water displacement in the pulvinus lead to shrinkage of the motor cells and provoke rapid bending and thus closure of the leaflets.^[^ ^17^, ^18^, ^19^, ^20^ ^]^

Movements and mechanisms similar to leaf unfolding can also be observed in the opening and closing of flowers. Certain flowers open and close only once, while others undergo this movement repeatedly before dying back.^[^ ^21^ ^]^ When closed, some flowers, such as morning glory, have pleated petals^[^ ^22^, ^23^ ^]^ whereas others, such as lily, show curved tepals.^[^ ^6^, ^24^ ^]^ Various mechanisms have been proposed to regulate opening and closure in different species, including changes in the tepal midrib,^[^ ^6^ ^]^ growth difference on the abaxial and adaxial side/local lamina expansion^[^ ^23^, ^25^ ^]^ and cell expansion.^[^ ^26^ ^]^ Reversible cell expansion can occur during multiple opening cycles, like in *Kalanchoe blossfeldiana*.^[^ ^27^ ^]^ The mechanism of flower opening in lilies has been a subject of controversy. Bieleski et al.^[^ ^6^ ^]^ indicated that the tepal midrib plays a crucial role in flower opening, while Liang and Mahadevan^[^ ^24^ ^]^ showed that opening can occur without the midrib. They indicated that differential growth along the tepal edges is responsible for flower opening, as edge wrinkling becomes visible at the onset of movement. Asymmetrically growing tissue can induce mechanical stress in a structure, followed by deformation and movement.^[^ ^28^ ^]^

**Figure 1:**
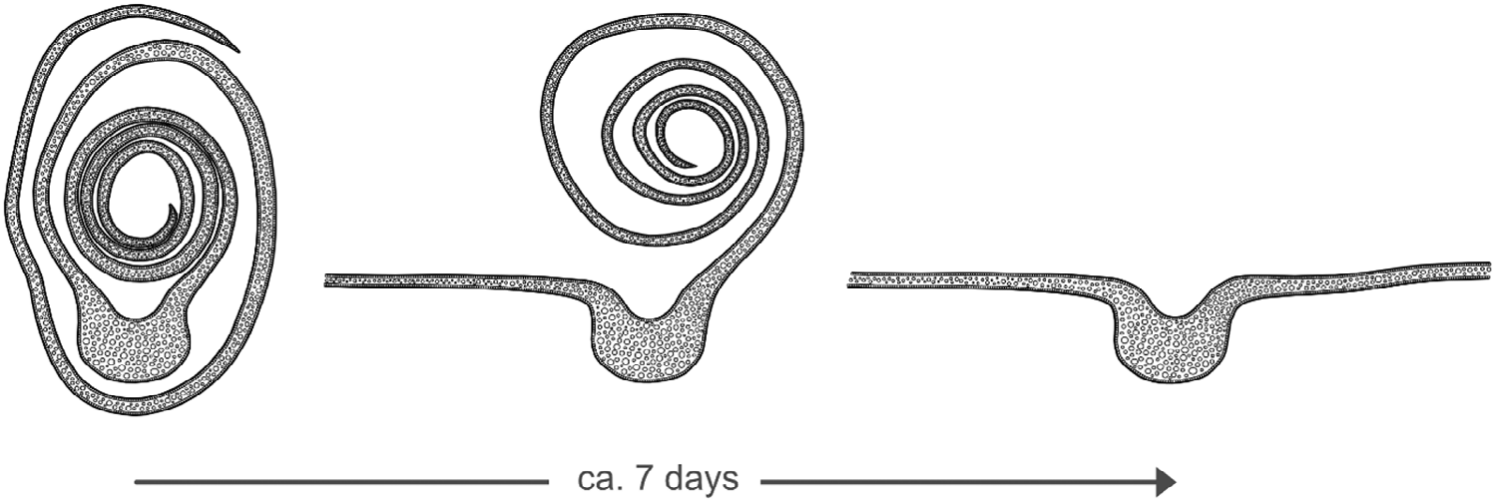
Schematic illustration of leaf unrolling in *Syngonium* *podophyllum*, showing the successive unrolling of the two leaf halves.

Unrolling leaves can be found in plants with peltate leaves, characterized by a stalk (petiole) attached to the center of the leaf blade, rather than the edge. This gives the leaf a shield‐like appearance, commonly seen in plants such as water lilies^[^ ^29^, ^30^ ^]^ and certain species of geraniums.^[^ ^29^, ^31^, ^32^ ^]^ Peltate leaves typically grow in a “rolled‐up” form, which promotes “unrolling” like in some floating leaves and *S. podophyllum* or Pilea peperomioides,^[^ ^16^, ^33^ ^]^ instead of unfolding along creases such as in leaves of trees.^[^ ^1^, ^34^ ^]^ Also, depending on the species, peltate leaves can either unroll their leaf halves simultaneously or subsequently.^[^ ^16^ ^]^ *S. podophyllum* leaves unroll their two leaf halves one after the other (**Figure** **1**).

We aim to better understand the unrolling process of peltate leaves and to clarify underlying mechanisms. Therefore, the present study concentrates on the mechanisms involved in peltate leaf unrolling, with a focus on the species *S. podophyllum* (Araceae) and deepens the understanding of the unrolling processes by modeling the uncurling in various ontogenetic phases in a selected plant species.

To this goal, we measured changes in anatomy, biomechanical properties (i.e., Young's modulus of the leaf lamina) as well as forces that are generated during unrolling. We propose a physical model for the opening process in leaves of *S. podophyllum* based on the mathematical models for nonlinear plate theory, which allow to describe very thin surfaces undergoing large deformations. Abstractly, the model will take into account dimensional as well as material properties of a rolled‐up leaf in order to predict its radius of curvature. Conversely, if the curvature is known, the model can be used for determining internal parameters which may be hard to determine experimentally.

To provide mathematical context, the proposed model is based on the work of Friesecke et al.,^[^ ^35^ ^]^ who rigorously derived a nonlinear, two‐dimensional plate theory—the Kirchhoff type plate model—from three‐dimensional, nonlinear elasticity theory. Their derivation, which relies on a notion of convergence for minimization problems, applies when the three‐dimensional elastic energy scales cubically with the plate's thickness (i.e., the leaf in our case) and when the material is homogeneous. A key tool in their approach is a geometric rigidity estimate, which allows to prove a precompactness result for the gradient of a given sequence of deformations. Before such convergence theorems were available, models were usually justified by expansion analysis approaches.^[^ ^36^ ^]^ Notably, the well‐known nonlinear Kirchhoff–Love Ansatz did not yield the correct theory later proved by Friesecke et al.^[^ ^35^ ^]^

Such a theory, however, still comes short to describing the unrolling of leaves, as the above theory assumes that the planar and undeformed state of the plate (or leaf) has no internal strain, i.e., no prestrain is present, which would forbid any kind of rolled‐up state to be energetically stationary in the absence of boundary conditions. Indeed, if a rolled‐up stage of the leaf is optimal, then applying a force to flatten it, would induce an internal strain (in the planar configuration).

These aspects have been successfully addressed in Schmidt (2007),^[^ ^37^ ^]^ which further extended the theory to allow for (1) prestain and (2) the material to be heterogenous in vertical direction. The latter permits the plate material to consist of an arbitrary amount of different, vertically stacked layers, each being homogenous. Therefore, our model is based on this further extended theory, as will be explained below.

**Figure 2:**
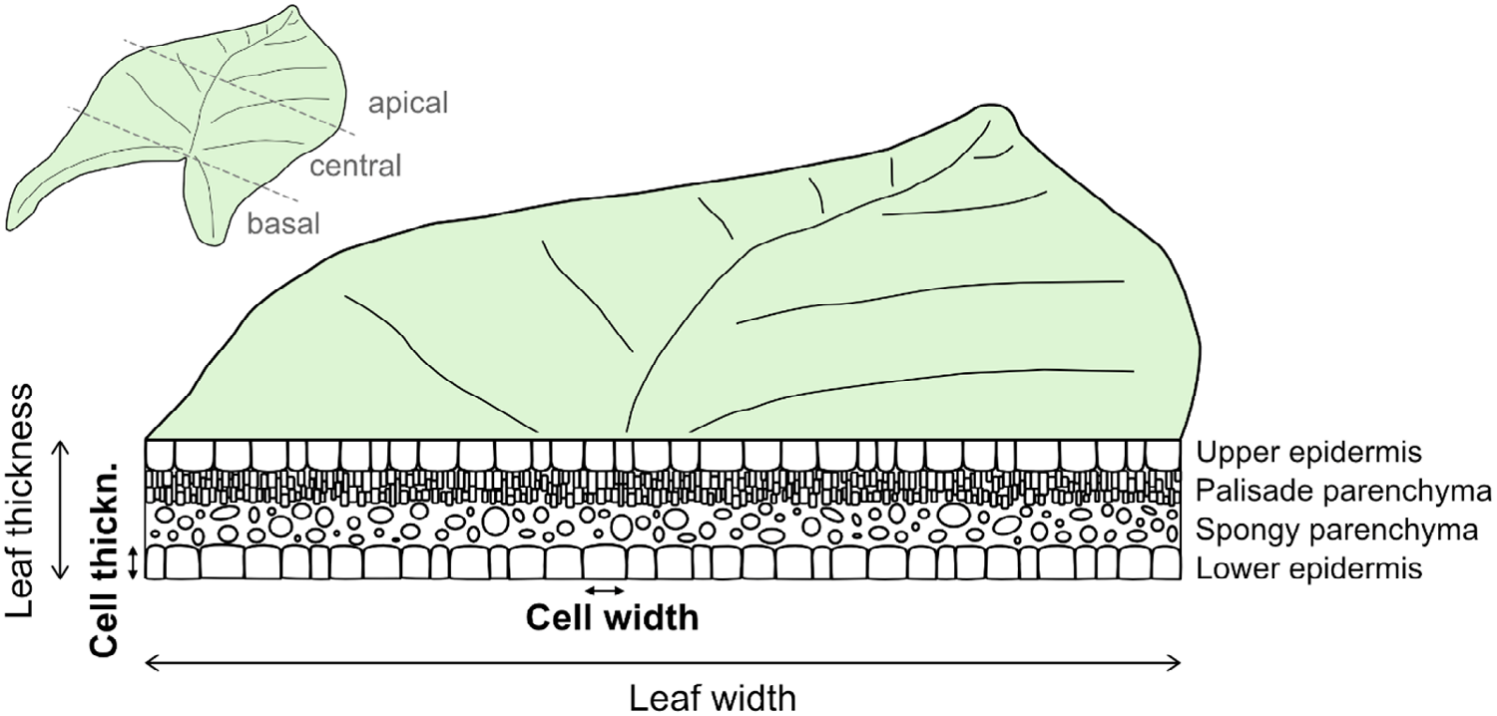
Modality scheme showing tissue distribution and leaf and cell orientation in a cross‐section in the central leaf region.

This approach allows to address the following questions:

1. Can the opening process in *S. podophyllum* leaves be adequately described by our model?
2. Is the process driven by anatomical changes in the whole leaf or by changes in specific tissues like the upper epidermis (located on the inner side of the folded leaf), the parenchymatous middle layer, or the lower epidermis? (cf. **Figure** **2**)
3. Is the process dominated by adaxial/abaxial differences of cell expansion, cell shrinkage, cell proliferation or cell apoptosis?

## 2 Results and Discussion

### 2.1 Anatomical Analyses

**Figure 3:**
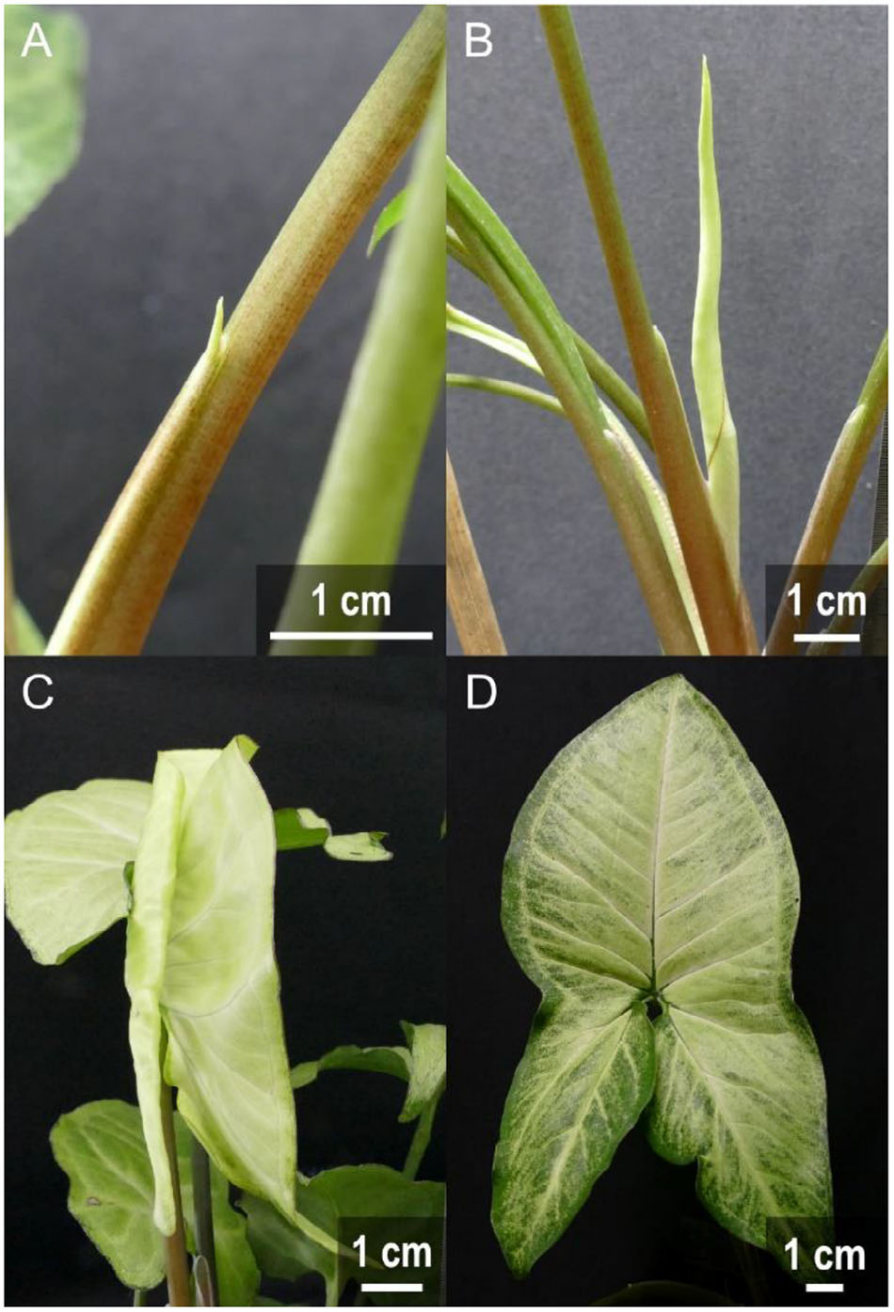
Macroscopic overview of the different leaf development stages of *S. podophyllum*. A) Stage S0–emergence from the leaf sheath. The leaf remains enclosed within the sheath, with only the tip visible. B) Stage S1—leaf unrolling imminent. C) Stage S2—intermediate stage of unrolling with one leaf half unrolled. D) Stage S3—unrolling completed.

**Figure 4:**
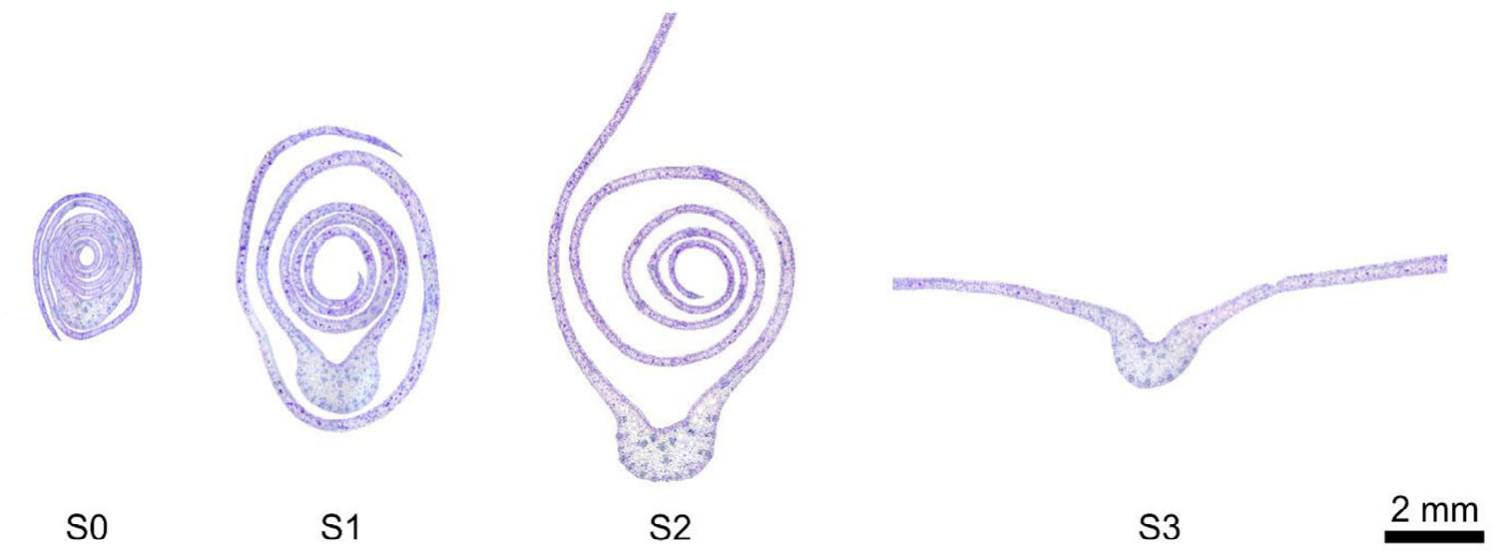
Anatomical cross‐sections at different stages during leaf development. S0–emergence from the sheath. S1–leaf unrolling imminent. S2—intermediate stage of unrolling with one leaf half unrolled. S3–unrolling completed. In stages S2 and S3, only a part of the lamina is displayed.

A key objective of the study was to determine the mechanisms responsible for the unrolling of peltate leaves in the species *Syngonium* *podophyllum* by anatomical analyses of different leaf stages (**Figure** **3**). Leaf development was classified into four different stages in which stages S1 and S2 constitute the onset and an intermediate stage of unrolling, respectively. At stage S0, the leaf emerges from the leaf sheath and at stage S3, the leaf completed unrolling. When rolled‐up, one leaf half is wrapped around the other one. This causes the outer half to unroll from the outside in, starting at the outermost area and moving toward the midrib. In contrast, the inner half unrolls from the innermost area to the outside (**Figure** **4**). For the outer half, the movement is analogous to “unrolling” a kitchen roll, whereas for the inner half, the movement is identical to “rolling out” a carpet.

**Figure 5:**
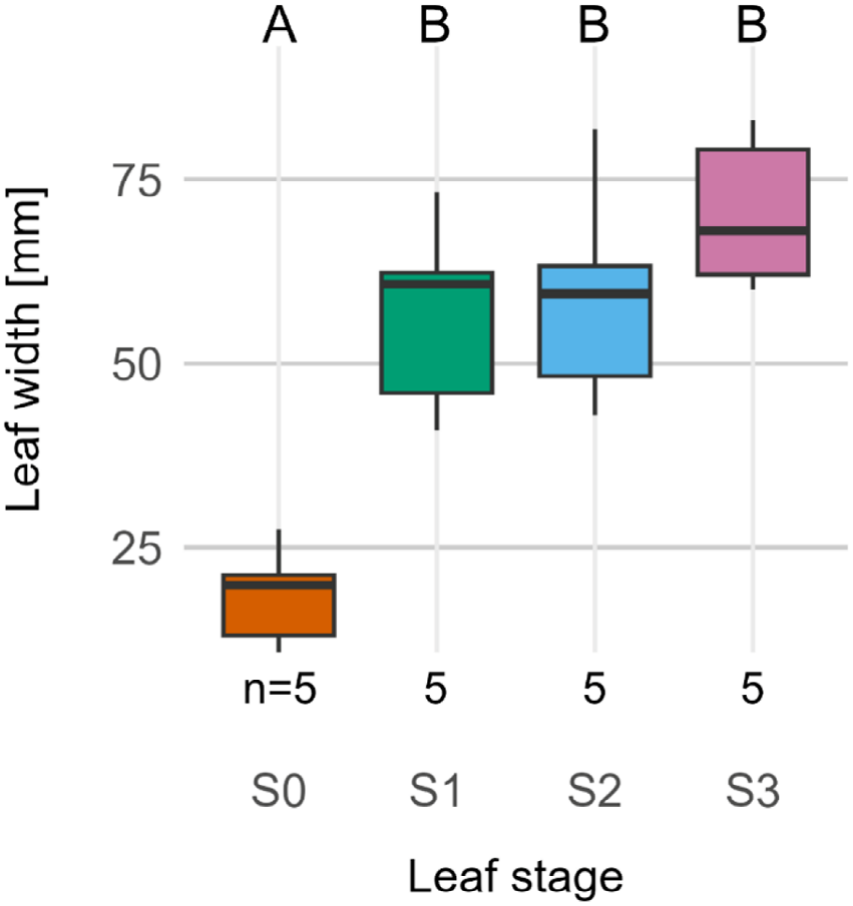
Leaf width at different stages during leaf development. S0—emergence from the sheath. S1—leaf unrolling imminent. S2—intermediate stage of unrolling with one leaf half unrolled. S3—unrolling completed. Upper case letters above the plot area indicate statistical significance (*p* < 0.05): groups that differ significantly have no common letter. Statistical method: one‐way ANOVA and Tukey HSD. Sample size: *n *= 5 per stage.

As can already be observed qualitatively in the anatomical cross‐sections (Figure 4) and is supported by the measurements (**Figure** **5**), the leaf significantly grows in width between stages S0 and S1. This is different from, e.g., banana leaves, which are practically fully grown when emerging from their sheaths.^[^ ^38^ ^]^ During the subsequent unrolling process (between stages S1 and S3), there is no significant increase in leaf width observed (Figure 5).

**Figure 6:**
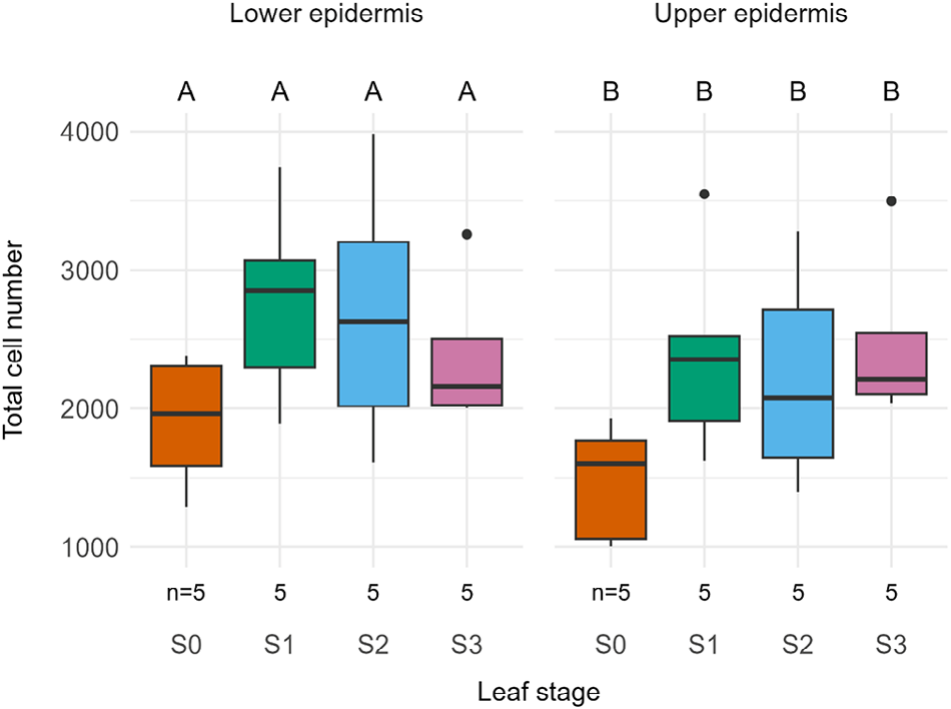
Total cell number across leaf width at different stages during leaf development in both the upper and lower epidermis. S0—emergence from the sheath. S1—leaf unrolling imminent. S2—intermediate stage of unrolling with one leaf half unrolled. S3—unrolling completed. Upper case letters above the plot area indicate statistical significance (*p* < 0.05): groups that differ significantly have no common letter. Statistical method: one‐way ANOVA. Sample size: *n* = 5 per stage.

Upon closer examination of the outermost tissues, specifically the upper and lower epidermis, total cell number across leaf width remains stable during leaf development. No increase in quantity was detected. Despite a generally lower total cell number in stage S0, no significant differences could be found compared to the other stages, neither in the lower nor in the upper epidermis (**Figure** **6**).

**Figure 7:**
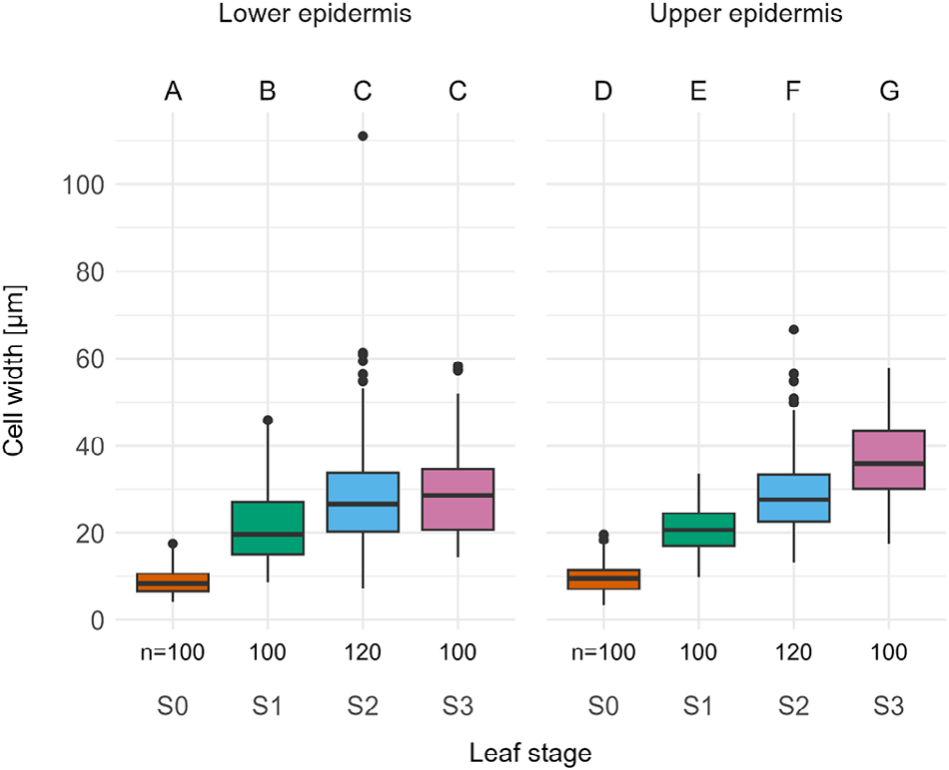
Cell width at different stages during leaf development in both the upper and lower epidermis. S0—emergence from the sheath. S1—leaf unrolling imminent. S2—intermediate stage of unrolling with one leaf half unrolled. S3—unrolling completed. Upper case letters above the plot area indicate statistical significance (*p* < 0.05): groups that differ significantly have no common letter. Statistical method: Kruskal–Wallis rank sum test and Dunn's test with Holm adjustment. Sample size: *n* = 100 for stages S0, S1, and S3, and *n* = 120 for stage S2.

**Figure 8:**
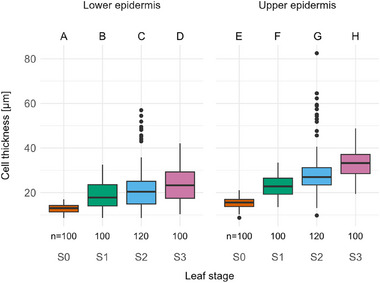
Cell thickness at different stages during leaf development in both the upper and lower epidermis. S0—emergence from the sheath. S1—leaf unrolling imminent. S2—intermediate stage of unrolling with one leaf half unrolled. S3—unrolling completed. Upper case letters above the plot area indicate statistical significance (*p* < 0.05): groups that differ significantly have no common letter. Statistical method: Kruskal–Wallis rank sum test and Dunn's test with Holm adjustment. Sample size: *n* = 100 for stages S0, S1, and S3, and *n* = 120 for stage S2.

Other than total cell number, cell dimensions alter while the leaf is developing. The lower epidermis cells show a tendency to grow both in (tangential) width and in (radial) thickness, hereafter referred to as width and thickness, respectively (see Figure 2). In the upper epidermis, however, cells significantly expand in width and in thickness between every stage (**Figures** **7** and **8**). Cross‐comparison of the upper and lower epidermis showed that cell width differed significantly in stage S3 and cell thickness differed significantly in every stage except for stage S1.

These results collectively indicate that epidermal cells grow throughout the developing leaf, but cell expansion predominantly occurs in the upper epidermis during leaf development. In areas with a higher curvature (like the inner coil of the leaf), an equal amount of expansion causes a greater effect than in less curved areas (like the outer coil of the leaf). This phenomenon is explained by thermoelastic curvature models and theories of nonlinear dynamics in initially curved beams.^[^ ^39^, ^40^, ^41^, ^42^ ^]^ Based on this, we suggest that the inner epidermis experiences a larger change in angle for each unit of length added, compared to the outer epidermis. Since this “shift” happens in the direction of leaf with (cf. Figure 2)—which is identical to the direction in which the leaf is unrolling—we also propose that leaf unrolling in *S. podophyllum* occurs as a result of changes in the upper epidermis, specifically driven by cell expansion rather than cell proliferation. Expanding epidermal cells contribute to leaf unfolding in other families, such as Gramineae, Cyperaceae, Juncaceae, Liliaceae, Amaryllidaceae, and Commelinaceae, particularly evident in the latter, where the entire upper epidermis is involved in the unfolding through cell expansion.^[^ ^38^ ^]^ Unfolding through cell expansion has as well been observed in some grass leaves, but they do so in a different way. They have specialized cells, the bulliform cells, that develop in the creases of the lamina and provoke reversible unfolding upon changes in turgor pressure.^[^ ^8^, ^9^ ^]^

Already when emerging from the sheath, the leaf tissues and structures of *S. podophyllum* are fully developed. After that, the whole leaf grows mainly via cell expansion till it unrolls. During unrolling, the leaf roughly maintains its overall width, while the expanding upper epidermal cells drive the movement. Whether cell wall properties in the different tissues change during unrolling has not been specifically investigated. However, the directional dependence of the lamina's Young's modulus throughout the process,^[^ ^16^ ^]^ together with the cell wall being the primary determinant of intrinsic tissue stiffness and shape,^[^ ^43^ ^]^ suggest that wall properties of *S. podophyllum* during unrolling are anisotropic and may, therefore, also play a role in the process. The importance of cell wall properties in leaf development is further supported by observations in later stages of tomato leaf primordia development, where asymmetric stiffening of cell walls contributes to the directional flattening and growth of the lamina.^[^ ^44^ ^]^

In addition to comparing cell width and thickness across stages, we assessed whether cell dimensions and growth is isodiametric by determining the aspect ratio (AR) of cell width and thickness. In stage S0, cells in both the upper and lower epidermis are anisodiametric in a way that their thickness exceeds their width. As the leaf develops, the anisodiametry of the epidermal cells shifts to the other side, indicating that their width increases more than their thickness (**Table** **1**), causing anisotropic growth.

**Table 1:** Cell aspect ratio (AR) ± SD of the upper and lower epidermis across leaf stages.

	S0	S1	S2	S3
AR upper epidermis []	0.62 ± 0.17	0.94 ± 0.27	1.03 ± 0.30	1.11 ± 0.21
AR lower epidermis []	0.69 ± 0.21	1.20 ± 0.42	1.37 ± 0.47	1.26 ± 0.33

S0—emergence from the sheath. S1–leaf unrolling imminent. S2—intermediate stage of unrolling with one leaf half unrolled. S3—unrolling completed. Sample size: *n*  =  100 for stages S0, S1, and S3, and *n* = 120 for stage S2.

Anisotropic growth induces residual stress, which tissues mitigate by adapting to a minimal‐energy configuration, often through mechanisms such as buckling or bulging.^[^ ^44^ ^]^ No buckling or bulging is observed during leaf unrolling in *Syngonium podophyllum*—a continuous process—and we propose that the growth stress is concentrated in the upper epidermis (due to cell expansion), and that this stress distribution drives the unrolling of the lamina as the system's energy‐efficient configuration.

### 2.2 Leaf Curvature during Unrolling

**Figure 9:**
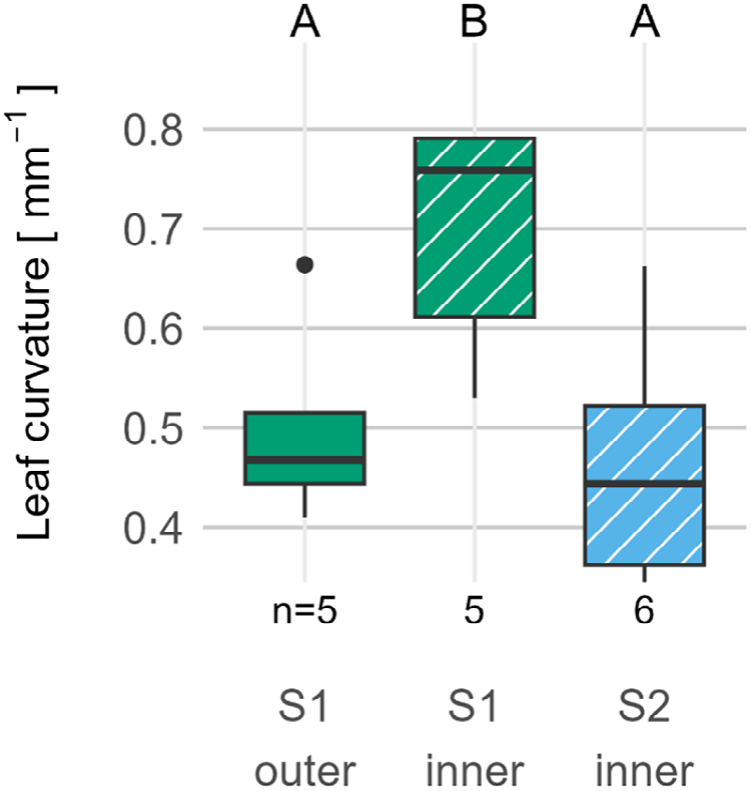
Leaf curvature of inner and outer halves in the region that will unroll first. S1—leaf unrolling imminent. S2—intermediate stage of unrolling with one leaf half unrolled. Hatched boxplots indicate the same (i.e., inner) leaf half. Upper case letters above the plot area indicate statistical significance (*p* < 0.05): groups that differ significantly have no common letter. Statistical method: one‐way ANOVA and Tukey HSD. Sample size: *n* = 5 for stages S1 outer and S1 inner, and *n* = 6 for stage S2 inner.

Leaf curvature was measured during the stages relevant to unrolling (S1 and S2) in the region that is the first to unroll in order to investigate dynamics of unrolling initiation. Before unrolling begins, the inner half naturally has a greater curvature than the outer half. During unrolling of the outer half, the inner half already loosens, reducing its curvature till it shows a similar curvature to the outer half prior to its unrolling (**Figure** **9**).

We could show previously that the Young's modulus of the leaf lamina in *S. podophyllum* is relatively low during and increases only after unrolling is completed, which is probably advantageous to the unrolling process.^[^ ^16^ ^]^ In the context of turgor‐driven cell expansion, which was discussed above, the further increase in turgor pressure after leaf unrolling likely enhances leaf stiffness, resulting in a higher Young's modulus compared to earlier stages.^[^ ^16^ ^]^

### 2.3 Forces During Leaf Unrolling

**Figure 10:**
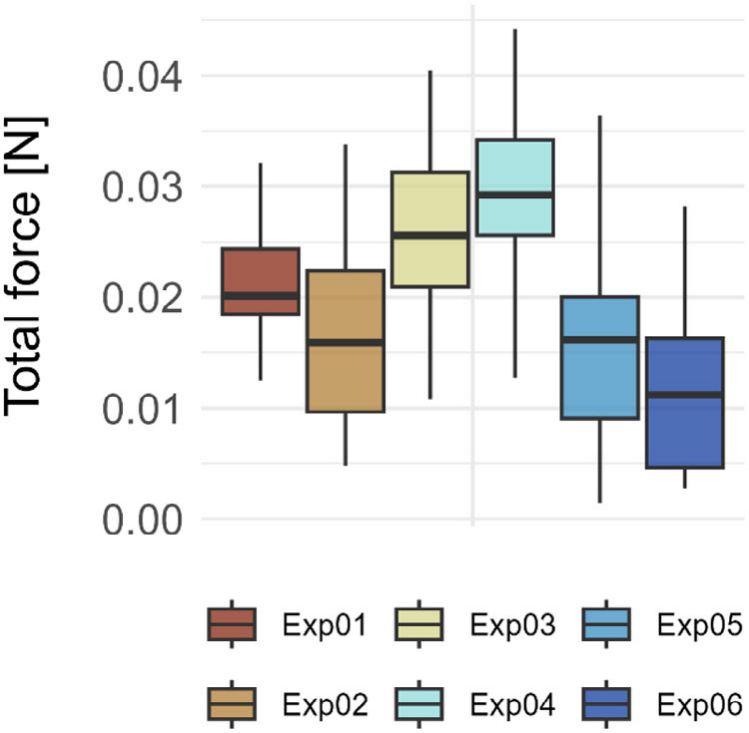
Total force generated during leaf unrolling, determined starting from 5% of the initial time of measurement.

To gain a deeper understanding of the unrolling process, forces occurring during unrolling were measured. The measured force is an outward‐directed force generated by the unrolling leaf. The leaves of *S. podophyllum* can develop forces up to 0.04 N (**Figure** **10**) while unrolling. However, force increase is not continuous: all experiments show that force drops may occur during the process of leaf unrolling (Supporting Information).

The developing force shows no correlation with watering and the measurement is not affected by simply entering the phytochamber. After being exposed to a period of drought, however, a sharp increase in force was observed shortly after watering (Video 06, Supporting Information).

These results indicate that water availability and thus turgor pressure are crucial for unrolling. The leaves unroll smoothly when watered on a regular basis but retard under drought stress. It has been shown previously, that water potential and turgor pressure regulate cell expansion and differentiation in developing grass leaves. When turgor pressure is low, for example due to drought stress, cell expansion and differentiation are inhibited.^[^ ^45^, ^46^, ^47^ ^]^ Similar to developing grass leaves, high turgor pressure seems to favor cell expansion and thus unrolling in leaves of *S. podophyllum*. In certain broadleaf trees, such as European oak, European beech and European hornbeam, the leaves experience a rapid osmotic adjustment during their early development and unfolding, a stage where drought tolerance is low.^[^ ^48^ ^]^ More extremely, some leaves refold under drought stress,^[^ ^10^, ^38^ ^]^ emphasizing how essential turgor pressure and water potential are to leaf unfolding.

Besides, turgor pressure is important in various plant parts and tissues, not only during leaf development. For example, in Caladium bicolor, another Araceae species, turgid parenchyma helps stabilize the petioles.^[^ ^49^ ^]^ More broadly, high turgor pressure plays a crucial role in maintaining the rigidity of parenchyma‐dominated tissues (e.g.,^[^ ^50^, ^51^ ^]^).

*Syngonium podophyllum* leaves can develop forces in a range of 0.02 and 0.04 N with forces up to 0.044 N during unrolling. We assume similar forces for both leaf halves during unrolling due to same tissues and similar respective curvature at the onset of unrolling, although forces could only be measured for the inner half due to the leaves’ configuration. To our knowledge, no research has been conducted on force development during unrolling leaves or flowers thus far. *Syngonium podophyllum* is a tropical species and grows self‐supporting in early stages before it matures to a hemi‐epiphytic climbing plant that attaches to its substrate via adhesive roots.^[^ ^52^ ^]^ Tropical rainforests have one of the highest plant diversities of any climate zone, with vegetation growing in multiple layers.^[^ ^53^ ^]^ In this densely packed environment, leaves must be able to unfold or unroll beside neighboring, probably even touching foliage to compete for space and sunlight. Based on our results, we assume that forces in the millinewton range during leaf unrolling are sufficient for this purpose. Though not directly related to leaf unrolling, the tendrils of *Passiflora caerulea*, which are modified inflorescences,^[^ ^54^, ^55^ ^]^ can develop forces ranging between 0.006 and 0.14 N, when they grasp a support and start free coiling in order to pull the plant stem closer to the substrate.^[^ ^56^ ^]^ The Venus flytrap *Dionaea muscipula*, whose lobes are modified leaves,^[^ ^57^ ^]^ has been thoroughly investigated in terms of trap closure (e.g.,^[^ ^58^, ^59^ ^]^). Forces of 0.15 N can arise when the prestrained lobes close.^[^ ^60^ ^]^

### 2.4 Multilayer Bending Model

In this section, we formulate the mathematical model to describe the observed curvature based on the model used in Schmidt (2007)^[^ ^37^ ^]^ and compare the results to a classical Timoshenko model approach. Additionally, we will derive a formula to obtain a layer mismatch from a prescribed radius for the Schmidt model that entails insights about the relationship between the radius and the layer mismatch. The derivation of the differential to formulate the mathematical model is described in the Experimental Section.

In the presence of heterogeneous layers, sudden expansion or contraction of either layer induces a curving strain and, since the leaf thickness is assumed to be small and the top and bottom layers are each homogeneous, Schmidt (2007)^[^ ^37^ ^]^ showed that the layer attains an energetically stable state when its curvature κ* satisfies:

(1) $$\kappa^{*}=\underset{\kappa \varepsilon R^{+}}{arg\;\min}{\bar{Q}}_{2}\left(\kappa \cdot {\left(1,0,0\right)}^{T}\right)$$

Here, Q̅_2_ is the dimensionally reduced linearized energy density at the identity, which depends on the material properties, layer properties, and the mismatch between layers, as constructed below. We describe the layer sheets as isotropic, homogeneous, elastic sheets. That is, let −0.5 ≤ *t* < 0.5 be the rescaled height within the leaf and let *λ* and *µ* be the Lamé parameters of each layer. We define

(2) $$Q_{2}\left(t,\;G\right)=2\mu \;{\left|sym\left(G\right)\right|}^{2}+\frac{2\mu \lambda }{2\mu +\lambda }{\left(tr\;G\right)}^{2}$$

where |*sym(G)*|^[^ ^2^ ^]^ and *tr G* denote the squared Frobenius norm of the symmetric part of G and the trace of a strain tensor *G*, respectively.

**Figure 11:**
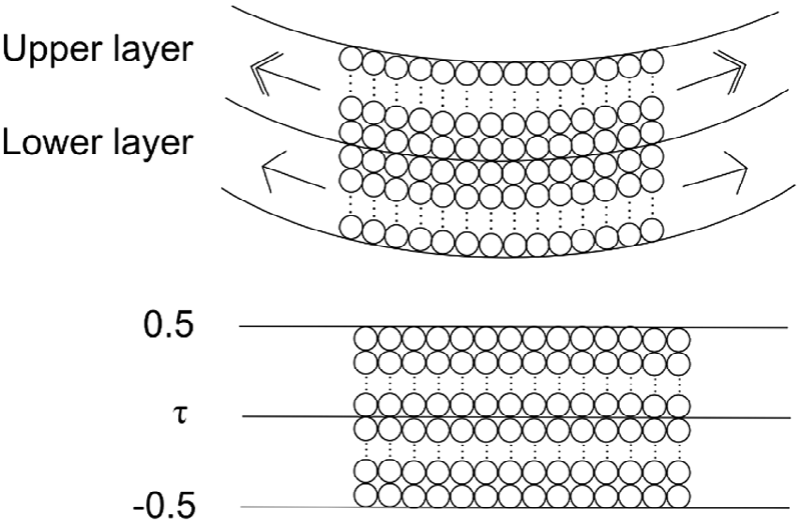
Schematic illustration of a simplified leaf for the mathematical model. The upper diagram shows the leaf in a rolled‐up state, where cells are idealized as circles and additional cell layers are indicated by dots. Arrows illustrate the expansion of these layers over time, with a double arrow emphasizing more significant expansion. The lower diagram depicts the leaf in its unrolled state. For the purposes of the mathematical model, the leaf's thickness is rescaled to 1, positioning it between −0.5 and 0.5, with *τ* representing the middle surface's location.

In our two‐layer setup, the bottom layer occupies [−0.5, *τ*], and the top layer occupies [*τ*, 0.5] (**Figure** **11**).

In particular, we let the constant material matrices *M*_bot_ and *M*_top_ denote the matrix representation of *Q_2_* specifically for the respective isotropy constants given by the Young's modulus *E = E(λ, µ)* and the Poisson's ratio *ν = ν(λ, µ)* for the respective layer.

**Figure 12:**
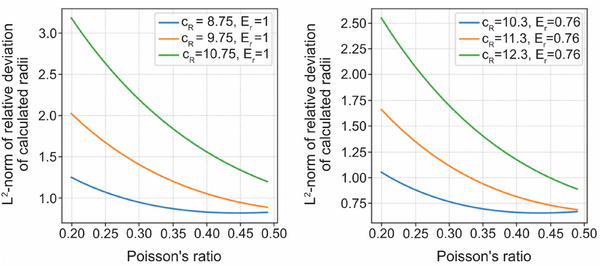
Sensitivity analysis of the mathematical model with respect to the assumed Poisson's ratio of 0.40. The squared *L* ^2^‐norm of the relative deviations across all samples is plotted against different values of the Poisson's ratio, under fixed parameters *c*_R_ and *E_r_*.

Based on our previous study on biomechanics of *S. podophyllum* leaves,^[^ ^16^ ^]^ a mean Young's modulus of 5.96 N mm^−2^ was used for the leaf in the model. Assuming isotropy, the results of the model suggest that this value effectively represents the properties of a leaf. The Poisson's ratio was taken from literature, which reports values between 0.3 and 0.5 as common for plant tissues (e.g.,^[^ ^61^, ^62^ ^]^). A value of 0.4 was considered appropriate for the model,^[^ ^63^ ^]^ which is corroborated by a sensitivity analysis (**Figure** **12**).

As shown in Schmidt (2007)^[^ ^37^ ^]^ the dimensionally reduced linearized energy functional at the identity is then given by

(3) $${\bar{Q}}_{2}\left(F\right)=\min_{A\;\in \;R_{\mathrm{sym}}^{2\times 2}}\int_{-1/2}^{1/2}Q_{2}\left(t,\;A+t\;F+B^{\prime }\right)\;dt$$

where B’ ϵ $R_{\mathrm{sym}}^{2\times 2}$ represents the dimensionally reduced layer mismatch. Specifically, in 3D elasticity, a prestrain is usually realized via a multiplicative perturbation, such that for a given deformation gradient *F*, the resulting energy is given by

(4) $$W_{0}(x_{3},F\left(Id+hB\left(x_{3}\right)\right)$$

where usually *W*_0_ is frame indifferent and has a single energy well at SO(3); the group of rotation matrices. The intuition behind this multiplicative perturbation, is that in classical plasticity theory, a deformation can be decomposed into an elastic part *F_e_*, i.e., a reversible deformation of the material and a plastic irreversible part *F_p_* such that *F* = *F_e_F_p_*. So, the above can be understood as fixing the plastic part to *Id* + *hB* allowing stretching and compressing. *B*′ is then obtained from *B* by omitting the last row and the last column.

To compute *Q̅_2_* explicitly, let ℳ(t) be the matrix representation of *Q_2_* (4), to define the following vertical averages:

(5) $$M_{1}=\underset{-\frac{1}{2}}{\int^{\frac{1}{2}}}M\left(t\right)\mathrm{dt},\;\;\;M_{2}=\underset{-\frac{1}{2}}{\int^{\frac{1}{2}}}t\;M\left(t\right)\mathrm{dt},\;\;\;M_{3}=\underset{-\frac{1}{2}}{\int^{\frac{1}{2}}}t^{2}\;M\left(t\right)\mathrm{dt}\;\;\;$$

It can be shown that

(6) $${\bar{Q}}_{2}\left(f\right)={\left(f-\;f_{0}\right)}^{T}\;M_{0}\left(f-\;f_{0}\right)+c$$

where $M_{0}=M_{3}-M_{2}M_{1}^{-1}M_{2}$ and $f_{0}=M_{0}^{-1}\left(M_{2}M_{1}^{-1}b_{1}-b_{2}\right)$,

 where b_1_, b_2_ ϵ $R^{3}$ are given by

(7) $$b_{1}=0,\;\;\;\;\;b_{2}=\left(\frac{1}{2}\tau^{2}-\frac{1}{8}\right)M_{bot}b_{bot}\left(e_{1}+e_{2}\right)-\left(\frac{1}{8}-\frac{1}{2}\tau^{2}\right)M_{top}b_{top}\left(e_{1}+e_{2}\right)$$

Here *b*_top_ *, b*_bot_ ϵ **ℝ** describe the upper and lower layer mismatch respectively and directly correspond to the dimensionally reduced layer mismatch via $B_{\mathrm{bot}}^{\prime }=b_{\mathrm{bot}}Id$. Also *e_i_* refers to unit vectors and $M_{bot}$ and $M_{top}$ is just M(±0.5) respectively. Hence, minimizing *Q̅_2_(кe_1_)* is equivalent to obtaining a predicted optimal curvature of

(8) $$\kappa^{*}=arg\;\min_{\kappa \varepsilon R}\left(\alpha^{2}\kappa +2\beta \kappa +\gamma \right)$$

where $\alpha =e_{1}^{T}M_{0}e_{1},\;\beta =e_{1}^{T}M_{0}f_{0}$

 and a radius r is obtained through

(9) $$r=\frac{d}{h}\kappa$$

where *d* (in millimeters) denotes the leaf thickness and *h* (dimensionless) represents the aspect ratio, defined as the ratio of leaf thickness to leaf width.

Since the layer mismatch is unknown in our setting, we compute it via an explicit formula for a prescribed radius of curvature. We assume a symmetric mismatch, i.e., *b*_bot_ = −*b*_top_, and, to simplify the formula, we assume that both layers share the same Poisson's ratio, while their Young's modulus is *E*_bot_ and *E*_top_, respectively. Denote by $M_{\mathrm{iso}}^{\left(1\right)}$ the theoretical material matrix (i.e., the matrix representation of *Q_2_*) specifically for *E* = 1 [N mm^−2^] and $\nu_{*}\;\varepsilon \;\left(0.1/2\right)$, in this case, we arrive at

(10) $$b_{\mathrm{bot}}=-b_{\mathrm{top}}=\frac{d}{h}\;\frac{e_{1}^{T}\;M_{0}\;e_{1}}{e_{1}^{T}M_{iso}^{\left(1\right)}\left(e_{1}+e_{2}\right)}\;\frac{2}{E_{\mathrm{top}}+E_{\mathrm{bot}}}\;\frac{1}{\frac{1}{4}\;-\;\tau^{2}}\;\frac{1}{r_{\mathrm{measured}}}$$

It is important to note that *M*_iso_ is specifically derived from *E* = 1 N mm^−2^, such that we may explicitly view the dependence of *b*_bot_ on the third factor from the right in the equation above, which would otherwise be absorbed into *M*_iso_. This factor is dimensionless, as the unit still resides in the given material matrix. More specifically, we have for a dimensionless factor *c* > 0,

(11) $$M_{\mathrm{iso}}^{\left(cE\right)}=c\cdot M_{\mathrm{iso}}^{\left(E\right)}$$

where $M_{iso}^{\left(E\right)}$ is the material matrix specifically for *E* and ν_*_. In the equation above we made use of this scaling via c = $\frac{E_{\mathrm{top}}+E_{\mathrm{bot}}}{2}$.

As we now have outlined a theory allowing us to describe the optimal state of a multilayer given a layer mismatch, the question arises, how we come up with a layer mismatch. As this theory has been used to describe MOVPE grown nanotubes, we will outline this case first without going into too much detail, as this has been thoroughly analyzed: Consider a crystalline substrate, idealized as a perfectly rigid boundary condition, with bulk lattice parameter *a*_sub_. When an ultrathin film whose bulk lattice parameter is *a_i_* is epitaxially deposited on the substrate, the film is compelled to adopt the in‐plane spacing of the substrate. Once the composite is removed, the film relaxes toward its own lattice parameter, thereby possibly inducing a bending moment. Thus defining

(12) $$\alpha_{i}=\frac{a_{i}}{a_{\mathrm{sub}}}$$

we may (in 3D, before going over to the limit) model a prestrain as a plastic deformation $F_{p}=\alpha_{i}^{-1}Id$. Finally, we can obtain a layer mismatch via the Ansatz α_i_ = 1 + *h*β_i_ as well as a simple Taylor expansion:

(13) $$\begin{array}{ccc} F_{p} & = & \alpha_{i}^{-1}Id={\left(1+h\beta_{i}\right)}^{-1}Id=\left(1-h\beta_{i}+O\left(h^{2}\right)\right)Id\\ & & =\left(1+hb_{i}\right)Id+O\left(h^{2}\right) \end{array}$$

Since the error is of order two, it vanishes in the limit, thus we obtain the following relation between the lattice constant and layer mismatch, same as in Schmidt (2007):^[^ ^37^ ^]^

(14) $$\alpha_{i}=\frac{1}{1+hb_{i}}$$

Next, we need to discuss how the layer mismatch for the model is obtained from our cell measurements in the leaf. The measurements consist of mean cell numbers $\bar{N}$ as well as mean cell widths *W̅* for both the top (upper) and bottom (lower) layer (epidermis and parenchyma, respectively) at the fully unrolled stage, referred to as S3, as well as rolled‐up and semi‐rolled‐up stages S1 and S2 (**Table** **2**).

**Table 2:** Mean values ± SD of cell characteristics relevant for the multilayer bending model.

	S1	S2	S3
Cell width upper epidermis [µm]	20.8 ± 5.4	29.1 ± 9.9	36.6 ± 9.4
Cell width lower epidermis [µm]	21.8 ± 8.8	29.1 ± 13.9	29.6 ± 10.6
Total cell number upper epidermis	2392 ± 737	2222 ± 773	2478 ± 602
Total cell number lower epidermis	2768 ± 714	2688 ± 943	2390 ± 525

S1—leaf unrolling imminent. S2—intermediate stage of unrolling with one leaf half unrolled. S3—unrolling completed.

For example, for the model we will refer to

(15) $${\bar{W}}_{\mathrm{upper},S1}$$

as the mean values of cell width of the top layer at time stage S1.

Our approach is to define the layer mismatch based on the following ratios

(16) $$\emptyset_{\mathrm{upper},S1,S3}=\frac{{\bar{N}}_{\mathrm{upper},S1}\cdot {\bar{W}}_{\mathrm{upper},S1}}{{\bar{N}}_{\mathrm{upper},S3}\cdot {\bar{W}}_{\mathrm{upper},S3}}$$

(17) $$\emptyset_{\mathrm{lower},S1,S3}=\frac{{\bar{N}}_{\mathrm{lower},S1}\cdot {\bar{W}}_{\mathrm{lower},S1}}{{\bar{N}}_{\mathrm{lower},S3}\cdot {\bar{W}}_{\mathrm{lower},S3}}$$

(18) $$\emptyset_{\mathrm{upper},S2,S3}=\frac{{\bar{N}}_{\mathrm{upper},S2}\cdot {\bar{W}}_{\mathrm{upper},S2}}{{\bar{N}}_{\mathrm{upper},S3}\cdot {\bar{W}}_{\mathrm{upper},S3}}$$

(19) $$\emptyset_{\mathrm{lower},S2,S3}=\frac{{\bar{N}}_{\mathrm{lower},S2}\cdot {\bar{W}}_{\mathrm{lower},S2}}{{\bar{N}}_{\mathrm{lower},S3}\cdot {\bar{W}}_{\mathrm{lower},S3}}$$

between the former and latter time stage in the subindex, i.e., from S1 to S3 or from S2 to S3.

We then define a layer mismatch as a relaxed version of the MOVPE‐layer mismatch while treating ∅ as a layer mismatch, i.e we propose the following relation:

(20) $$\frac{1}{1+hc_{R}B}=\emptyset \;\Leftrightarrow B=\frac{\frac{1}{\emptyset }\;-\;1}{c_{R}h}$$

where $c_{R}\in R^{+}$ is a relaxation factor that can be freely chosen. Again, for reference, Schmidt (2007)^[^ ^37^ ^]^ used the relation

(21) $$\frac{1+hb_{\mathrm{top}}}{1+hb_{\mathrm{bot}}}=\frac{\alpha_{\mathrm{bot}}}{\alpha_{\mathrm{top}}},\;\;i.e.\;\frac{1}{1+hb_{\mathrm{bot}}}=\alpha_{\mathrm{bot}},\;\;\mathrm{and}\;\frac{1}{1+hb_{\mathrm{top}}}=\alpha_{\mathrm{top}}$$

where *α* refers to the lattice constants of the homogeneous materials used there. For stages S1 and S2, the following leaf properties were relevant to the model: leaf curvature, thickness of the upper layer (i.e., upper epidermis and palisade parenchyma), and thickness of the lower layer (i.e., lower epidermis and spongy parenchyma). From these properties, the middle surface *τ* and plate thickness *h* were determined (**Table** **3**).

**Table 3:** Leaf properties relevant for the multilayer bending model.

Leaf stage	Sample number	Radius of curvature *r*_M_ [mm]	Thickness upper layer [mm]	Thickness lower layer [mm]	Middle surface *τ* [/]	Plate thickness h [mm]
S1	01	1.89	0.057	0.109	0.156	0.0027
S1	02	1.32	0.061	0.143	0.203	0.0028
S1	03	1.27	0.051	0.171	0.269	0.0053
S1	04	1.26	0.051	0.131	0.222	0.0039
S1	05	1.64	0.061	0.145	0.203	0.0033
S2	01	1.87	0.070	0.106	0.101	0.0029
S2	02	1.51	0.055	0.106	0.161	0.0030
S2	03	2.46	0.063	0.143	0.193	0.0048
S2	04	2.08	0.054	0.149	0.234	0.0031
S2	05	2.90	0.050	0.136	0.230	0.0023
S2	06	2.88	0.054	0.131	0.209	0.0040

S1—leaf unrolling imminent. S2—intermediate stage of unrolling with one leaf half unrolled.

The leaf properties, the middle surface and the plate thickness were used to calculate a theoretical radius of curvature. Then, its relative percentage deviation to the measured counterpart (i.e., the measured radius of curvature of the respective leaf) was determined (**Table** **4**).

**Table 4:** Results of the model given by the data of Table 2.

Leaf stage	Sample number	Ratio_bot	Ratio_top	*b_bot*	*b_top*	Calculated radius of curvature *r*_C_ [mm]	Relative deviation [%]
S1	01	0.855	0.549	5.463	26.536	1.23	−34.69
S1	02	0.855	0.549	5.395	26.206	1.64	24.18
S1	03	0.855	0.549	2.799	13.598	2.08	64.58
S1	04	0.855	0.549	3.812	18.517	1.51	19.94
S1	05	0.855	0.549	4.536	22.034	1.65	0.96
S2	01	1.105	0.713	−2.869	12.186	1.81	−3.12
S2	02	1.105	0.713	−2.803	11.906	1.76	16.79
S2	03	1.105	0.713	−1.761	7.481	2.37	−3.81
S2	04	1.105	0.713	−2.671	11.345	2.54	22.34
S2	05	1.105	0.713	−3.663	15.560	2.31	−20.34
S2	06	1.105	0.713	−2.071	8.798	2.20	−23.74

S1—leaf unrolling imminent. S2—intermediate stage of unrolling with one leaf half unrolled.

To concisely present the following results, we fixed *c*_R_ = 8.9 and selected the elastic moduli *E*_top_  =  5.96 N mm^−^^2^ and *E*_bot_ = 4.53 N mm^−^^2^. Choosing *E*_bot_ slightly lower than *E*_top_ significantly reduced the variance in the differences between the measured and model‐predicted radii across our sample set. The value *c*_R_ = 8.9 is chosen to provide an appropriate centering of the predicted radii, yielding a mean deviation from the measured values of less than 0.01 mm. It should be emphasized that the results reported below are sensitive to the parameter choices outlined above. A more detailed discussion of this dependency is provided in the subsequent Poisson's ratio sensitivity analysis.

Given the set of measured radii and calculated radii of curvature, referred to as *r*_M_ and *r*_C_, the correlation coefficient

(22) $$cor\left(r_{C},r_{M}\right)=\frac{Cov\left(r_{C},\;r_{M}\right)}{\sqrt{Var\left(r_{C}\right)}\sqrt{Var\left(r_{M}\right)}}$$

as well as the mean and variance of differences were calculated in order to evaluate the model (**Table** **5**).

**Table 5:** Relevant statistics on the calculated radii of curvature *r*_C_ in relation to the measured radii of curvature *r*_M_.

Statistics	Value
Correlation coefficient	0.60
Mean of differences	−0.0035
Variance of differences	0.23

The multilayer bending model successfully predicts the correct order of magnitude for the measured radii of curvature. The results confirm the expected behavior that the measured and calculated radii of curvature are of the same order of magnitude, as the parameter *c*_R_​ can be freely adjusted.

A more compelling question is whether, and to what extent, variations in the predicted radii of curvature correspond proportionally to variations in the measured radii of curvature. In other words, we seek to determine whether there is a linear relationship between the two. This relationship can be assessed using the correlation coefficient.

While we do not formally prove that the correlation coefficient is the optimal statistical measure in this context—given that the statistical properties of the underlying dataset remain unknown—it provides a useful indication of the strength of the association. The correlation coefficient of 0.60 (within the interval [−1,1]) suggests a moderate positive linear relationship (Table 5). Considering that our analysis is based on averaged cell characteristics, this result is more than satisfactory. It confirms that the measured leaf properties (Table 3) contain essential information to predict the leaf radius of curvature and the model produces a set of calculated radii of curvature that capture the distribution of measured radii of curvature within this set moderately well. Notably, the correlation coefficient cor(*r*_C_,*r*_M_) is independent of *c*_R_. Indeed, revisiting the derivation of the model reveals that *c*_R_ introduces a factor in front of both the lower and upper layer mismatch, which in turn creates a factor in front of *f*_0_, *β*, and finally *κ*, under which the correlation coefficient is known to be invariant.

Examining individual sample results, we observe deviations ranging from 35% to +65%. These deviations fall within the expected margin of error due to the averaging of cell characteristics. Given that the two‐dimensional model is derived as an asymptotic approximation assuming vanishing leaf thickness, it is reasonable to expect that greater leaf thickness leads to reduced model accuracy. Additionally, the inability to measure the Young's modulus separately for the upper and lower layers may further contribute to discrepancies, particularly for leaves with a relatively thick lower layer.

In the sensitivity analysis of the mathematical model (Figure 12), *E*_r_ denotes the ratio $\frac{E_{\mathrm{bot}}}{E_{\mathrm{top}}}$, for which we considered the canonical value *E*_r_ = 1 (motivated by our measurements), as well as a slightly reduced value *E*_r_ = 0.76 as explained above. For each choice of *E*_r_, the corresponding value of *c*_R_ was selected to ensure that the mean difference between measured and predicted radii vanishes, resulting in *c*_R_ = 9.75 and 11.3 respectively. Additionally, we included the shifted values *c*_R_ ± 1 for comparison. The results indicate that selecting a Poisson's ratio of 0.4 appears to yield a good fit.

Finally, the formula reveals an anti‐proportional relationship between the calculated radius of curvature and layer mismatch. The term involving the Young's moduli indicates that the model effectively treats the limiting plate as an isotropic material with an average Young's modulus given by

(23) $$\frac{E_{\mathrm{top}}+E_{\mathrm{bot}}}{2}$$

This estimation aligns with expectations based on the model's asymptotic formulation. Additionally, the dependence on *τ* suggests that an uneven distribution of layer thickness must be compensated by a greater layer mismatch to produce the same radius of curvature.

The model's formulation also allows for an explicit expression in terms of the Young's modulus under the assumption *E* = *E*_top_ = *E*_bot_. This implies that, given the cell characteristics and the leaf radius of curvature, one could, in principle, infer material parameters. However, applying this approach to the current dataset would be of limited interest, as our prior selection of *c*_R_ ​ already ensures that the approximation is well within acceptable accuracy.

We also formulated a Schmidt based model with four layers, distinguishing between the epidermis layer and parenchyma layers, since the parenchyma layers have an explicitly smaller Young's modulus than the epidermis layers.^[^ ^64^ ^]^ Such a model yielded very similar results and no improvements with a correlation coefficient of 0.58.

In contrast to the exploratory model above, the classical Timoshenko formula for a two layer setup is given by^[^ ^64^, ^65^, ^66^ ^]^

(24) $$r=\frac{d_{1}^{4}+4\chi d_{1}^{3}d_{2}+6\chi d_{1}^{2}d_{2}^{2}+4\chi d_{Q1}d_{2}^{3}+d_{2}^{4}\chi^{2}}{6\varepsilon \left(1+\nu \right)d_{1}d_{2}\left(d_{1}+d_{2}\right)\;}$$

Here *d*_1_ and *d*_2_ are the thickness of the bottom and top layer, respectively, $\chi =\frac{E_{2}\;}{E_{1}}$ is the quotient of the Young's moduli, ν is the Poisson's ratio and ɛ is the in‐plane biaxial strain, i.e., if is the lattice constant of the first layer then α_1_ = α_0_(1 + ɛ).

Since the formula above is not formulated for a layer mismatch, we first use the following translation $\alpha_{0}=\frac{1}{1+hB_{0}}$ and $\alpha_{1}=\frac{1}{1+hB_{0}}\left(1+\varepsilon \right)$ before inserting our relaxed layer mismatch as derived above for the Schmidt model. We chose the same Young's moduli as for the Schmidt model, as well as a parameter *c_R_* that centers the mean (**Table** **6**).

**Table 6:** Results of the model using the Timoshenko approach, given by the data of Table 2.

Leaf stage	Sample number	Ratio_bot	Ratio_top	*b_bot*	*b_top*	Calculated radius of curvature *r_C_* [mm]	Relative deviation [%]
S1	01	0.984	0.926	5.993	29.112	1.35	−28.75
S1	02	0.984	0.926	5.918	28.751	1.76	33.68
S1	03	0.984	0.926	3.071	14.918	2.20	73.77
S1	04	0.984	0.926	4.182	20.315	1.62	28.41
S1	05	0.984	0.926	4.976	24.173	1.78	8.66
S2	01	1.009	0.962	−3.147	13.370	1.76	−5.99
S2	02	1.009	0.962	−3.075	13.062	1.68	11.44
S2	03	1.009	0.962	−1.932	8.207	2.24	−9.05
S2	04	1.009	0.962	−2.930	12.447	2.37	14.28
S2	05	1.009	0.962	−4.018	17.070	2.16	−25.50
S2	06	1.009	0.962	−2.272	9.653	2.07	−28.23

S1–leaf unrolling imminent. S2–intermediate stage of unrolling with one leaf half unrolled.

The deviations are slightly worse than from the Schmidt approach, with, e.g., a correlation coefficient of 0.47 compared to 0.60 (**Table** **7**).

**Table 7:** Relevant statistics on the calculated radii of curvature *r*_C_ in relation to the measured radii of curvature *r*_M_ using the Timoshenko approach.

Statistics	Value
Correlation coefficient	0.47
Mean of differences Variance of differences	0.008 0.29

Nevertheless, the Timoshenko based model provides an alternative approach to the Schmidt approach which is Kirchhoff‐based with a cubic energy scaling. This cubic scaling arises because the Kirchhoff‐based model neglects sheer and focuses primarily on bending. In contrast, the Timoshenko‐based model additionally includes sheer effects such that the resulting energy scaling with respect to the plate thickness is linear rather than cubic. Such shear is particularly relevant in the regime where the plate thickness is no longer negligible, thus making the Timoshenko approach more suitable for thicker plates compared to the Kirchhoff‐based model. More analysis will have to be done to determine the generally more suitable model, again as our data above is very limited.

Ultimately, the parameter *c*_R_ plays a crucial role in this model, which arises because we know how to describe the internal stress induced by a mismatch in the atomic lattice structure in the case of homogeneous materials, but we do not yet fully grasp how an identical mechanism of layer expansion and contraction causing internal stress—but this time at a macroscopic level—compares in this regard.

Additionally, the homogeneous isotropic description may lead to limitations in some settings, as leaves are inhomogeneous structures, with, e.g., parenchyma being the least stiff plant tissue^[^ ^67^ ^]^ Further, due to the constraints of sample preparation, it was not possible to track changes in curvature for the same leaf across different stages. Instead, the model relies on approximations using mean values from leaves at different stages. Increasing the sample size would likely have improved the results to feed the model.

**Figure 13:**
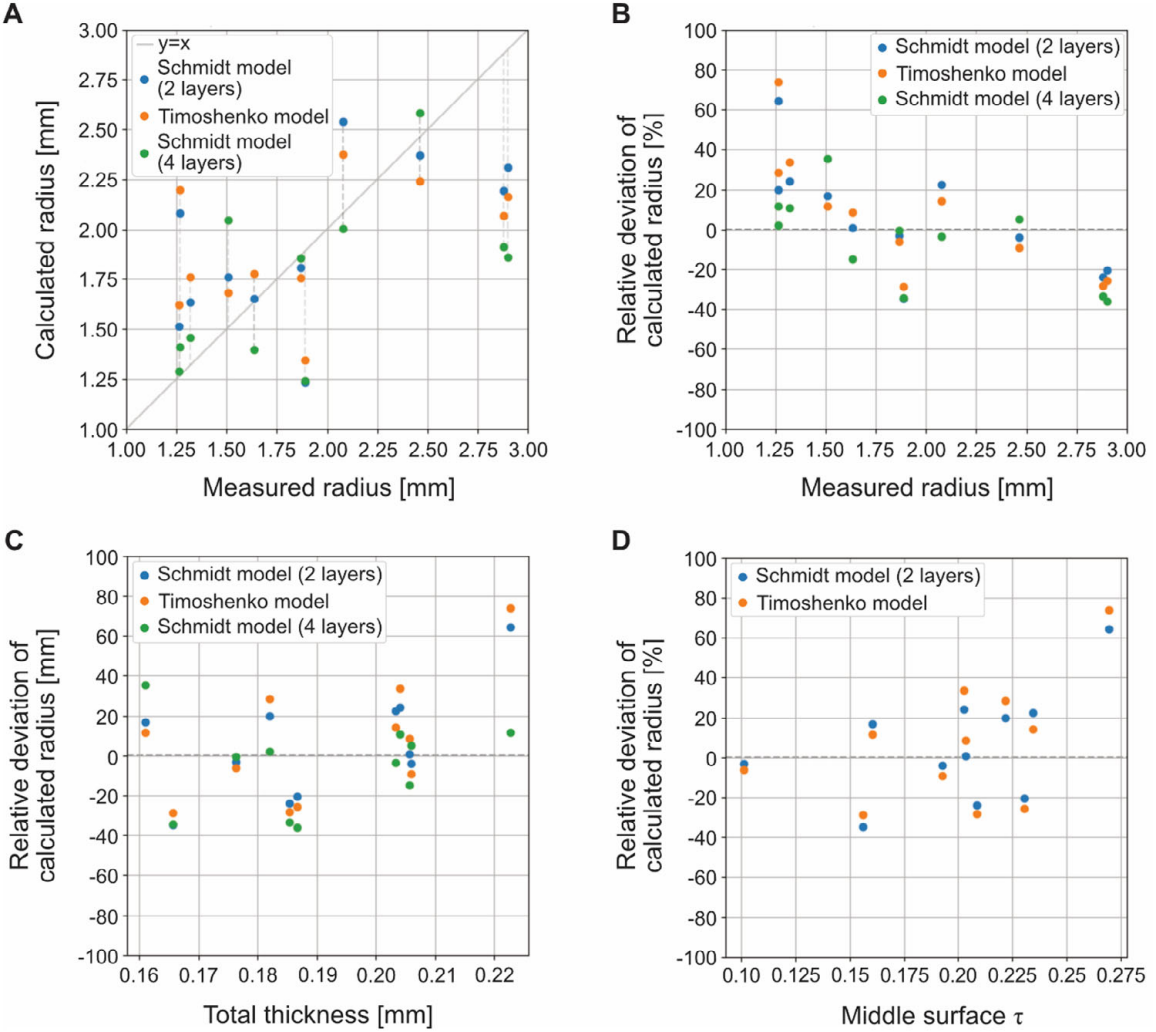
Accuracy evaluation of the mathematical model. A) Absolute comparison of calculated radii. B) Relative comparison of calculated radii. C) Comparison of calculated radii versus total thickness. D) Comparison of calculated radii versus middle surface *τ*.

In general, the model overestimated small radii and underestimated large radii (**Figure** **13**). This outcome is not unexpected, given that the model relies on average layer mismatches, which correspond to a moderate radius. For the total thickness and the middle surface, there is a slight though inconclusive trend. This is a positive sign, as these two parameters are available on a per‐sample basis. However, given the lack of a larger sample size, it is too early to draw conclusions.

Despite the above, the results are promising and suggest that further investigations are warranted to refine and challenge the model for understanding the leaf unrolling in peltate leaves.

## 3 Conclusion

Leaf unrolling in *S. podophyllum* occurs successively in the two leaf halves and appears to be mainly driven by cell expansion. As for other plant tissues and movements, turgor pressure plays an important role in cell expansion and thus in initiating unrolling. Forces in the millinewton range are sufficient for the leaves to unroll in an environment with surrounding foliage.

As a first approximation, the mathematical model can predict changes in curvature of an unrolling leaf. The model may be of interest to assess changes in curvature for other structures with two or more layers. This includes but is not limited to other species that “unroll” or markedly bend their leaves or scales, as snap‐tentacles present in some *Drosera* species and to bending pine cone scales.

Moreover, direct mechanical testing of epidermal layers, along with in vivo monitoring of cell turgor pressure during leaf development, hold strong potential to deepen our understanding of leaf unrolling in peltate species.

## 4 Experimental Section

### Species Cultivation

Specimens of *S. podophyllum* were cultivated in the Freiburg Botanic Garden at an average temperature of 25 °C and 70% relative air humidity. The plants were watered so that the soil remained slightly moist.

### Thin Sections of Leaves

Cross‐sections were prepared for anatomical analyses. For this purpose, four leaf stages, based on Modert et al. (2024),^[^ ^16^ ^]^ were defined. At stage S0, the leaf begins to emerge from the sheath. Stage S1 represents the folded leaf just before unrolling. At stage S2, the leaf is unrolling, with one half open and one half closed. Finally, at stage S3, the leaf is fully unrolled.

Leaves were cut to size by a razor blade and prepared for embedding in Technovit 7100 (Kulzer GmbH, Hanau, Germany). Embedding consisted of fixation, dehydration, infiltration, polymerization and blocking. Cut to size leaf samples were transferred into formaldehyde acetic acid alcohol (FAA) for fixation overnight. Then, the dehydration series was performed as follows:

70 % 2‐Propanol overnight
90 % 2‐Propanol for 2 h, samples ventilated
100 % 2‐Propanol for 30 min, samples ventilated
100 % 2‐Propanol for 1 h, samples ventilated

The samples were transferred into the preinfiltration solution, consisting of 2‐Propanol and base solution in a 1:1 ratio. Preinfiltration took place overnight and samples were ventilated. Next, the infiltration solution was prepared according to the manufacturer's instructions: 1 g of hardener I was dissolved in 100 mL base liquid. Infiltrating samples were briefly ventilated and then stored overnight at 4 °C. After infiltration, polymerization solution was prepared according to the manufacturer's instructions: 1 mL of hardener II was mixed with 15 mL preparation solution. The samples were taken from the infiltration solution and remains of the solution were removed with paper towels. Then, the samples were transferred into a Teflon embedding mold (Histoform, Kulzer GmbH, Hanau, Germany) and polymerization solution was added. The samples were aligned so that cross‐sections were generated during subsequent cutting. Polymerized samples were blocked to remove them from the embedding mold. For this purpose, Technovit 3040 Powder and Technovit Universal Liquid (Kulzer GmbH, Hanau, Germany) were mixed at a ratio of approximately 2:1, poured onto the polymerized samples and small plastic blocks were mounted. After hardening, the sample blocks were removed by carefully loosening them with pliers.

Sample cutting was realized by an automatic rotary microtome (fully automatic rotary precision microtome CUT 6062, V3.11, SLEE medical GmbH, Mainz, Germany). Sample blocks were clamped into the microtome and aligned to the blade (hard metal knife, 16 cm long, profile D, steel assembly, Leica Biosystems Nussloch GmbH, Nussloch, Germany). The sections were transferred onto microscope slides wetted with distilled water and dried on a hot plate at 60 °C.

Afterwards, along the lines of Gerlach (1984),^[^ ^68^ ^]^ the sections were stained with toluidine blue 0.05% (Toluidine Blue O, Chroma‐Gesellschaft Schmid & Co., Stuttgart, Germany) in distilled water. Staining solution was applied for 30 seconds and rinsed with distilled water. The microscope slides were air‐dried overnight before permanent sealing with mounting medium (Entellan new rapid mounting medium for microscopy, Merck KGaA, Darmstadt, Germany) and a cover slip.

Stained samples were analyzed under a transmitted light microscope (Olympus BX61, Olympus Corporation, Tokyo, Japan) using the software Olympus Cell^P (version 2.6, Olympus Corporation, Tokyo, Japan) and images of different magnifications were taken by a microscope camera (Olympus DP71, Olympus Corporation). Background impurities or errors in image composition by the microscope software were edited in an image editing software (GIMP, version 2.10.38).

### Anatomical Analyses

**Figure 14:**
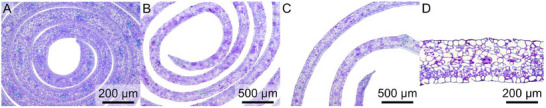
Representative detail images used for anatomical analyses, shown for each leaf stage. A) Stage S0–emergence from the sheath. B) Stage S1—leaf unrolling imminent. C) Stage S2—intermediate stage of unrolling with one leaf half unrolled. D) Stage S3—unrolling completed.

To identify the mechanisms that are responsible for unrolling, anatomical changes were investigated during leaf development and, particularly, during leaf unrolling. Cell dimensions were determined, i.e., cell width and thickness, of the upper and the lower epidermis in the central region of the leaf (Figure 2). The cell dimensions were measured for five to six leaves at each stage, with 10 cells measured per leaf. In the rolled‐up leaf stages, the upper epidermis was located on the inner side of the coil, while the lower epidermis was on the outer side (**Figure** **14**). The central region was defined as the area extending from the petiole insertion point to the midpoint between this point and the apical tip.

Moreover, the total cell number of the upper and lower epidermis as well as leaf width of each stage were determined. More specifically, leaf width was considered as the distance from the left to the right margin, corresponding to the full length of the coil in the rolled‐up state. Cross‐sections from microtome cutting were used for all measurements except for total cell number in stage S3 (fully unrolled). In stage 3, fresh sections produced on a cryostat (SLEE Cryostat MEV, SLEE medical GmbH, Nieder‐Olm, Germany) were used. Leaves were cut into smaller samples using a razor blade. Samples were transferred onto custom‐built sample plates (technical department, Faculty of Biology II/III, University of Freiburg) and embedded in a water soluble medium (Tissue‐Tek O.C.T. Compound, Sakura Finetek, Torrance, USA). Sections were prepared using a blade suitable for cryostats (hard metal knife, 16 cm long, profile C, steel assembly, Leica Biosystems Nussloch GmbH).

### Leaf Curvature during Unrolling

**Figure 15:**
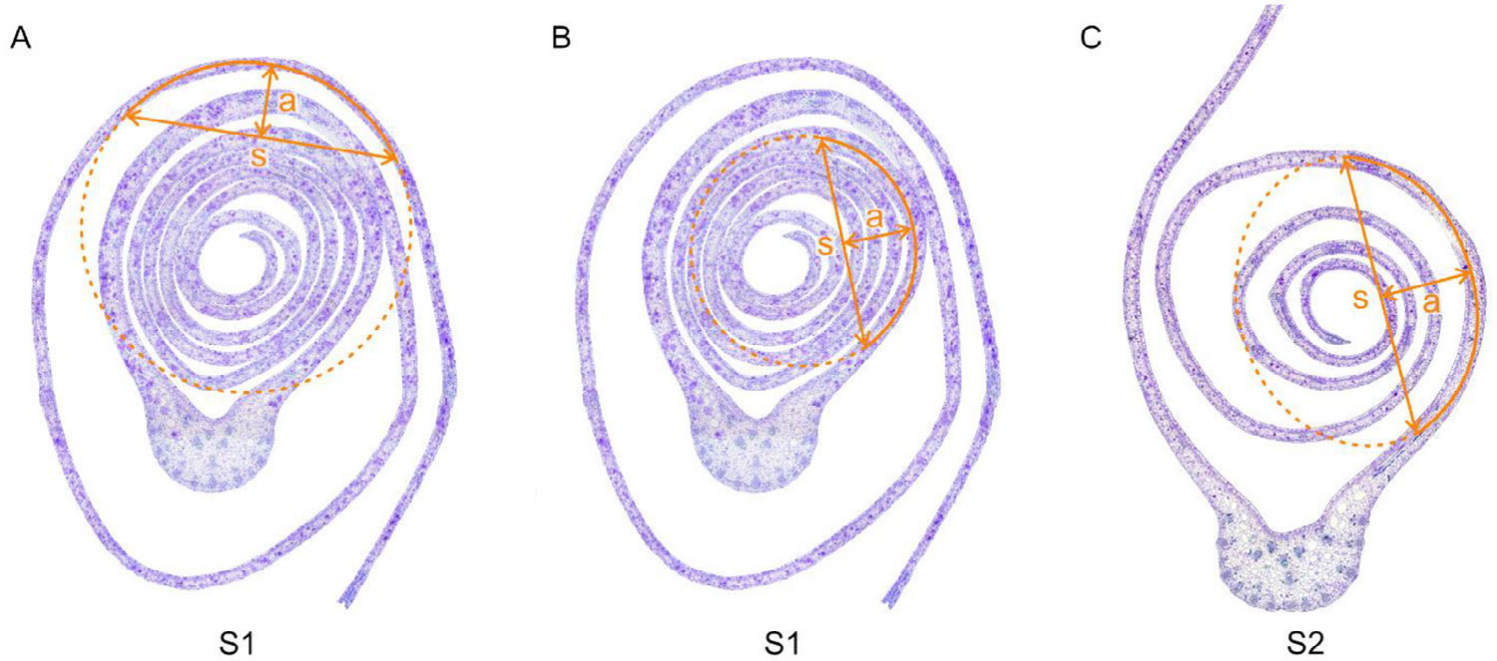
Method for determining leaf curvature. A,B) Stage S1 representing the onset of unrolling, with the outer and the inner half measured respectively. C) Stage S2 representing an intermediate phase of unrolling, with one leaf half open. s—chord length of the ellipse at the outermost point of the curve. a—curvature height at the apex of the curve perpendicular to chord length.

Leaf curvature was determined at the beginning of and during unrolling (stages S1 and S2) by means of the cross‐sectional pictures and an image processing software (ImageJ, version 1.54 g). Curvature was measured in the region unrolling next in the respective stage. An additional measurement was taken for stage S1 to be used in the multilayer bending model. Specifically, the curvature was measured on the inner half of the leaf near the mid‐rib. This is the region that unrolls first in stage S2. By measuring the curvature of the inner half at two different time points, the necessary data was obtained for the model (**Figure** **15**).

The curvature *K* is defined as the reciprocal of the radius of curvature *r*. The radius of curvature could be approximated by a circular arc:

(25) $$r=\frac{4a^{2}+s^{2}}{8a}$$

with *s*: chord length of the ellipse at the outermost point of the curve and *a*: curvature height at the apex of the curve perpendicular to chord length.

Measuring leaf curvature at various stages was one of the key parameters used to develop a model that describes leaf unrolling as the (un)bending of multiple layers (details in section “Multilayer Bending Model”).

### Forces During Leaf Unrolling

**Figure 16:**
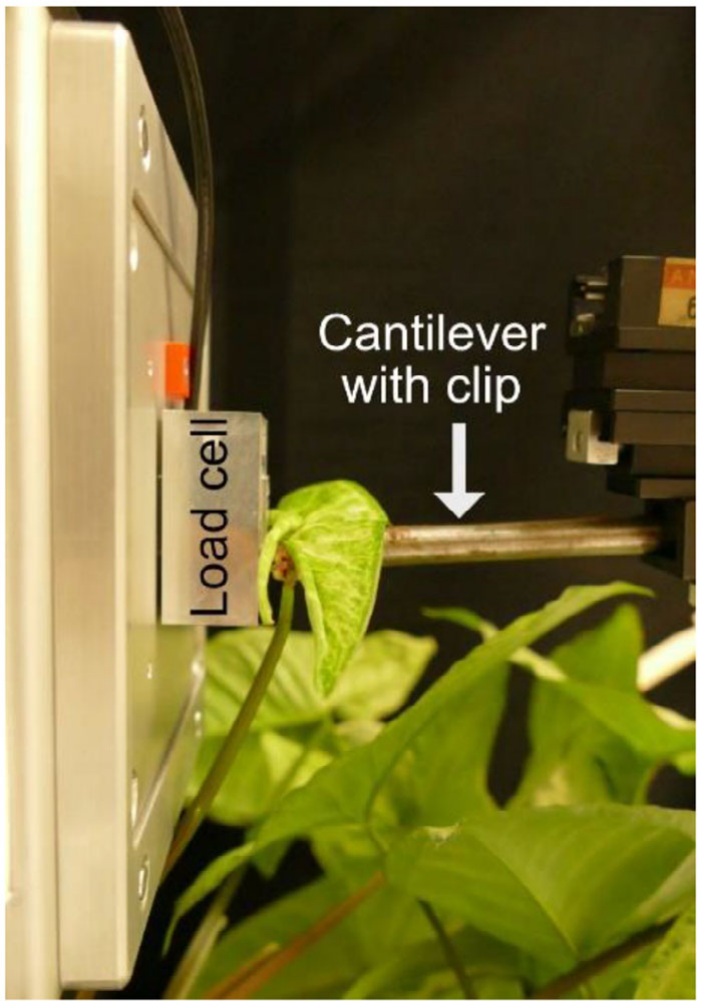
Side view of the experimental setup used to measure the forces during unrolling of *S. podophyllum* leaves in stage S2. The load cell is a three‐axis force sensor, and the cantilever with clip is mounted on a micromanipulator (right), allowing precise three‐dimensional positioning of the leaf in front of the load cell.

In addition to analyzing the changes in leaf curvature, the forces that occur during unrolling were measured. The measured force is an outward force exerted by the unrolling leaf that “pushes” against a load cell during the unrolling process (**Figure** **16**). The measurements were conducted in a phytochamber that maintained the same environmental conditions as those used for species cultivation, with 24‐h illumination for timelapse recordings. These measurements were specifically taken for stage S2 on the folded leaf half using a custom‐built setup created by the technical workshop (Faculty of Biology II/III, University of Freiburg). A three‐axis‐force sensor (K3D40 ±2 N, accuracy class 0.5%, ME‐Meßsysteme GmbH, Hennigsdorf, Germany) together with a measuring amplifier (GSV‐8DS SubD44HD, ME‐Meßsysteme GmbH) were integrated in the setup and measurements were recorded at an interval of 2 min by the manufacturer's software (GSVmulti, version 1.46.1 2020) while the leaf was unrolling.

Total force was calculated as a vector quantity with components in *x*, *y*, and *z* directions:

(26) $$F_{\mathrm{tot}}=\sqrt{{F_{x}}^{2}+{F_{y}}^{2}+{F_{z}}^{2}}$$

In addition, the unrolling leaf was recorded at an interval of 20 min by a digital camera (Panasonic Lumix DMC‐FZ1000, Panasonic Corporation, Kadoma, Japan). Timelapse videos were generated at a rate of 25 fps in a video editing software (DaVinci Resolve, version 19.0.3, Blackmagic Design Pty Ltd, South Melbourne, Australia). Similarly, videos were created from the plotted force data to synchronize and merge timelapse recordings with force measurements in one video.

### Multilayer Bending Model

By taking into account leaf characteristics such as changes in curvature over time and changes on the cellular level in the upper and lower epidermis, this work aims to mathematically describe the unrolling process.

The proposed model was derived from plate theory for heterogeneous multilayers,^[^ ^37^ ^]^ originally used to describe nanotubes fabricated using MOVPE‐grown (metal‐organic vapor‐phase epitaxy grown) homogeneous materials (see also^[^ ^69^ ^]^). These nanotubes curled due to a mismatch in the atomistic lattice of the constituent layers when the multilayer structure was released from the substrate. Mathematically, this model is obtained via dimension reduction of the corresponding three‐dimensional elasticity theory, resulting in a formulation suited for very thin surfaces. For a full formal derivation and mathematical justification, refer the reader to Schmidt (2007).^[^ ^37^ ^]^

In the present application, the model was adapted to a two‐layer configuration to represent the upper epidermis and palisade parenchyma as the top layer, and the spongy parenchyma as well as the lower epidermis as the bottom layer. Specifically, the model defines a middle surface of infinitesimal small thickness that separates these layers. Due to the challenges in obtaining direct measurements, both layers are assumed to be isotropic, with the Young's moduli determined experimentally from leaf samples. Additionally, it is important to note that, while the nanotubes in the original context curled upon release, the leaves unroll in the opposite manner. However, this is considered unproblematic as the model determines an energetically stationary point, which is independent of time. In other words, the least energy configuration does not depend on the motion of the thin surface.

The critical aspect of adapting the model for the present application is the accurate representation of the layer mismatch. Assuming that differences in cell numbers and cell growth between the two layers induce the curvature, one may define a macroscopic length scale variation for each layer. However, this does not directly correspond to a differential in an atomistic lattice structure required for the model. However, it is hypothesized that a macroscopic length scale variation calculated from cell growth measurements can be mapped to an atomistic lattice differential within the model by appropriately scaling this macroscopic length scale differential, keeping a proportional relationship but allowing for more flexibility.

### Statistical Analyses

Results were evaluated using R (version 4.4.1) and R Studio (version 2024.09.1).^[^ ^70^ ^]^ Data were checked for parametric distribution via Shapiro–Wilk normality test. The data for leaf width (*n* = 5 per stage), for total cell number across leaf width (*n* = 5 per stage) and for leaf curvature (*n* = 5 for stage S1 and *n* = 6 for stage S2) followed a parametric distribution (*p* > 0.05), while the data for cell width and cell thickness of both the lower and upper epidermis (*n* = 100 for stages S0, S1, and S3, and *n* = 120 for stage S2) showed a nonparametric distribution (*p* < 0.05). In case of parametric data—that is leaf width, total cell number across leaf width as well as leaf curvature—a one‐way ANOVA was performed to analyze significance levels. If significant differences were found (*p* < 0.05), a Tukey HSD test was used as a post hoc test to identify significantly different groups. In case of a nonparametric distribution—that is cell width and thickness of the lower and upper epidermis—a Kruskal–Wallis rank sum test was performed to analyze significance levels. If significant differences were found (*p* < 0.05), Dunn's test with Holm adjustment was used as a post hoc test to identify significantly different groups.

To process data for statistical analyses and for plotting, the following R packages were applied: *tidyr* for data rearrangement,^[^ ^71^ ^]^ *dplyr* for data filtration and extraction,^[^ ^72^ ^]^ *dunn.test* for statistical testing,^[^ ^73^ ^]^ *ggplot2* for plot generation,^[^ ^74^ ^]^ and *grid* for creation of a graphical element.^[^ ^70^ ^]^ Data analysis is presented as boxplots with interquartile range or as mean values ± standard deviation (SD).

## Conflict of Interest

The authors declare no conflict of interest.

## Supporting information



Supplemental Movie 1

Supplemental Movie 2

Supplemental Movie 3

Supplemental Movie 4

Supplemental Movie 5

Supplemental Movie 6

## Data Availability

All relevant data and resource can be found within the article and its supporting information.
